# Cosmetic wastewater treatment technologies: a review

**DOI:** 10.1007/s11356-022-23045-1

**Published:** 2022-09-22

**Authors:** Despina A. Gkika, Athanasios C. Mitropoulos, Dimitra A. Lambropoulou, Ioannis K. Kalavrouziotis, George Z. Kyzas

**Affiliations:** 1 Department of Chemistry, International Hellenic University, Kavala, Greece; 2 Department of Chemistry, Aristotle University of Thessaloniki, Thessaloniki, Greece; 3 School of Science and Technology, Hellenic Open University, Patras, Greece

**Keywords:** Cosmetics, Wastewater treatment technologies, Physical methods, Chemical methods, Biological methods, Green technologies

## Abstract

Over the past three decades, environmental concerns about the water pollution have been raised on societal and industrial levels. The presence of pollutants stemming from cosmetic products has been documented in wastewater streams outflowing from industrial as well as wastewater treatment plants. To this end, a series of consistent measures should be taken to prevent emerging contaminants of water resources. This need has driven the development of technologies, in an attempt to mitigate their impact on the environment. This work offers a thorough review of existing knowledge on cosmetic wastewater treatment approaches, including, coagulation, dissolved air flotation, adsorption, activated sludge, biodegradation, constructed wetlands, and advanced oxidation processes. Various studies have already documented the appearance of cosmetics in samples retrieved from wastewater treatment plants (WWTPs), which have definitely promoted our comprehension of the path of cosmetics within the treatment cycle; however, there are still multiple blanks to our knowledge. All treatments have, without exception, their own limitations, not only cost-wise, but also in terms of being feasible, effective, practical, reliable, and environmentally friendly.

**Graphical abstract:**

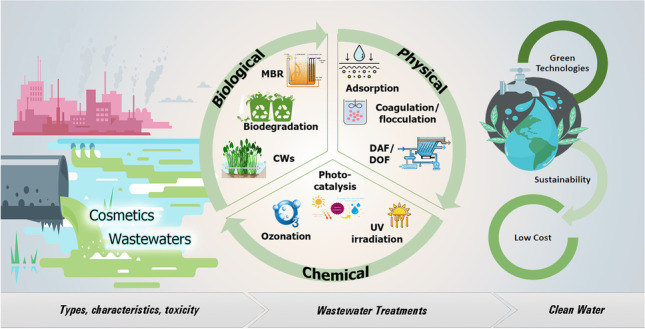

**Supplementary Information:**

The online version contains supplementary material available at 10.1007/s11356-022-23045-1.

## Introduction

During the last decades, the impact of chemical pollution has gained increased interest especially those industrial intermediates displaying persistence in the environment (Daughton and Ternes 1999). Industrial evolution has heavily burdened natural resources and has escalated the environmental pollution, uncovering noticeable climate changes (Yenkie 2019). As an example, the cosmetic industry produces over 3000 synthetic compounds such as antibiotics, painkillers, antidepressants, and contraceptives, which can be used to address the symptoms of various illnesses (Beiras 2021). Unfortunately, the continuous rise of the cosmetics’ production translates to increased production of waste (Bogacki et al. 2020) and industrial wastewater is one of the most significant pollution types (Kyzas and Mitropoulos 2021). It is formed by washing equipment and by-products using a mix of water, surfactants, and disinfectants, which means that the waste contains the substances found in the produced cosmetics (Bogacki et al. 2020). Anti-inflammatory painkillers such as ibuprofen (IBU), antibiotics such as sulfamethoxazole (SMX), and antidepressants such as fluoxetine (FLU) have been found in significant concentrations in aquatic environments (Beiras 2021). Thus, cosmetics thus pose the most immediate ecological risk compared to pharmaceuticals due to their heavy use for long periods of time and due to being intended for external use, which means they are not metabolized and end up unaltered into the environment [2]. In this regard, failing to remove such compounds while treating the wastewater is the main reason why both the cosmetics and pollutants end up in large quantities in the environment (Klaschka et al. 2013; Montes-Grajales et al. 2017).

In the spectrum of chemicals, pharmaceuticals and personal care products (PPCPs) are both pieces of the larger puzzle used in the cosmetic industry (Gar Alalm et al. 2016). PPCPs are used as preservatives of ingredients in cosmetics that pose the highest concerns and contain compounds such as contrast agents, hormones, preservatives, beta-blockers, sunscreen UV filters, anti-inflammatory drugs, soaps, disinfectants, and detergents. Such pollutants have been detected globally in aquatic environments (Liu and Wong 2013). They initially enter the wastewater and then get transferred in wastewater treatment facilities (Awfa et al. 2018; Thomaidi et al. 2017). Due to their extensive use and bioactive properties, PPCPs have received a lot of attention regarding their fate, which has been enabled by the recent progress in analytical science, allowing researchers to detect substances at trace levels (Wang et al. 2017). More specifically, the most frequently detected compounds so far were galaxolide (up to about 600 μg/L in influents and about 110 μg/L in effluents) (Klaschka et al. 2013) and tonalide, whose concentration reached about 90 μg/L (Chen et al. 2007; Klaschka et al. 2013). Other frequently encountered materials include triclosan, ranging between 20 and 100 ng/L in Spain (Díaz-Garduño et al. 2017), and an insect repellent found in concentration levels ranging between 5 and 2100 ng/L in the USA (Loraine and Pettigrove 2006) and South Korea (Kim et al. 2007). In addition, the substance benzophenone-3 (used in sunscreens) was found in treatment sites in the both the UK and the USA (Kasprzyk-Hordern et al. 2009; Trenholm et al. 2008) in high quantities.

Cosmetics can be broadly categorized as leave-on and rinse-off products. A leave-on product is expected to remain on a person’s skin for a long period of time; such products include but are not limited to perfumes, body and face creams, and deodorants, whereas rinse-off cosmetics are expected to be rinsed off shortly after use and include shampoos, soaps, shower gels, and toothpastes (Juliano and Magrini 2017). Personal care and cosmetics can be used externally on the skin, nails, hair, lips, etc., or internally, for example, for oral hygiene, including cleaning, anti-germ protection, fresh breath, maintenance, and improvement of appearance (Aranaz et al. 2018). Contrary to pharmaceuticals, PPCPs can be only consumed externally; thus, they are more likely to end up into the environment in large concentrations due to human activities, thus straining the environment (Bulloch et al. 2015; Ternes et al. 2004). It is noteworthy that another difference between PPCPs and pharmaceuticals is that large amounts of PPCPs can be directly introduced to the environment (Daughton and Ternes 1999). In addition cosmetics are of higher priority compared to pharmaceuticals due to their excessive use by larger groups of people for longer periods of time (Juliano and Magrini 2017).

Wastewater treatment facilities and sewage outfalls are the most prominent sources of PPCPs released in the environment (Luo et al. 2014; Tiwari et al. 2017). Various works have examined the potential approaches for the removal of PPCPs from wastewater, at a pace with the progress achieved in terms of monitoring and analyzing the processes (Chen et al. 2016; Fu et al. 2019; Junaid et al. 2019; Kar et al. 2020; Liu et al. 2020; Liu et al. 2021; Papageorgiou et al. 2019; Paucar et al. 2019; Yuan et al. 2020). The removal efficiency of personal care products from treatment facilities varies significantly, depending on the compound, biological treatment process employed, and technology used as well as operating conditions (Alvarino et al. 2015). Nevertheless, the removal of these contaminants before they can reach surface water has been complicated, because of their low-concentration levels and challenges in analyzing them (Oluwole et al. 2020).

Typical wastewater treatments comprise generally of an assortment of physicochemical and/or biological processes (Crini and Lichtfouse 2019). The physical processes are useful in extracting solids from wastewater, most often via screens and filters, while biological ones utilize small organisms to eliminate and break down harmful waste. Chemical processes are usually paired with physical processes to extract more complicated contaminants (Yenkie 2019). Most treatment facilities use a technology from each phase, although often, more than one is required for the successful elimination of pollutants, according to the purity goals, pollutant characteristics, and their concentration. Occasionally, some can be skipped as well (Yenkie 2019). (Hussein and Jasim 2021). It should be noted that all options have their unique advantages and limitations, cost-wise, as well as in terms of effectiveness, suitability, and environmental effects (Crini and Lichtfouse 2019). In the light of the above, it may be assumed that one only specific approach is not applicable for an effective treatment. A fusion of diverse processes overcomes this challenge.

This work aims to review the existing knowledge on the removal of cosmetics and offer a comparison of available approaches, summarizing their potential advantages and disadvantages. To this end, this review will further explore the detection of cosmetics in the environment and focus on the technologies used in treating cosmetic wastewater in an effort to assess the potential measures required to constrain the presence of cosmetics in the environment. It also briefly outlines economic analysis of the technologies in the problems related to waste management and discusses the recovery of water from wastewater and its re-use.

This work is structured as follows: The “Cosmetic treatment technologies” section will examine in detail the most significant characteristics of the currently used physicochemical and biological approaches, while offering an overview of the effectiveness thereof, current status, and challenges faced. The “Discussion” section contains a discussion of the research outcomes, while the review is concluded in the “Conclusions” section.

## Cosmetic treatment technologies

The environmental degradation rate can be limited or prevented through the adoption of a wide range of economic and sustainable treatment approaches. To this end, further research into more efficient, environmentally-friendly, and cost-effective treatment options is required, with the objective to degrade the complex particles into simpler ones (Bello et al. 2018). A typical large-scale cosmetic wastewater treatment approach consists of coagulation combined with dissolved air flotation, followed up by additional biological treatment. This is a highly efficient process, but still not adequate to fully remove dangerous micropollutants, such as polycyclic musk, UV filters, heavy metals, and microplastics.

Various alternate options have been explored, including advanced oxidation processes (AOPs), which results in the effective generation of strong oxidants, such as radicals. During advanced oxidation, the formulation of radicals is enabled by the presence of iron cations (Fenton’s process and its variations), during which a major issue is to ensure there is a steady availability of iron cations. The quantity of the cations in a solution is affected by many parameters, such as pH, the recovery efficiency of Fe^2^ ions from Fe^3+^, and the rate with which Fe^2+^ ions are released. This can be addressed by monitoring the Fe^2+^/Fe^3+^ ions ratio or by the regulated continuous flow of Fe^2+^ ions into the solution. Both solutions are plagued by multiple technical difficulties when practiced in reality. As a result, iron-based heterogeneous co-catalysts have been in the forefront of recent research. Such co-catalysts include Fe^0^ (metallic iron, zero-valent iron, ZVI), Fe_2_O_3_, and Fe_3_O_4_. Oxides coordinate surface sites of Fe^2+^ that bind with the pollutants and reduce them (Bogacki et al. 2020).

Cosmetic wastewater may display increased levels of chemical oxygen demand (COD, > 100,000 mg/L), biological oxygen demand (BOD), and total organic carbon (TOC). Furthermore, it is common to find high quantities of petroleum ether extract, organic nitrogen, and organic phosphorus. Bogacki et al*.* used ferric chloride in an attempt to reduce the COD. The results suggested an up to 64% reduction of COD, at a pH level of 6.0 (Jan et al. 2011). In another study, Marcinowski et al. investigated coagulation at the optimal ferric chloride dose of 200 mg/L, leading to a COD reduction of about 39% (to 792 mg/L) (Marcinowski et al. 2014).

**Fig. 1:**
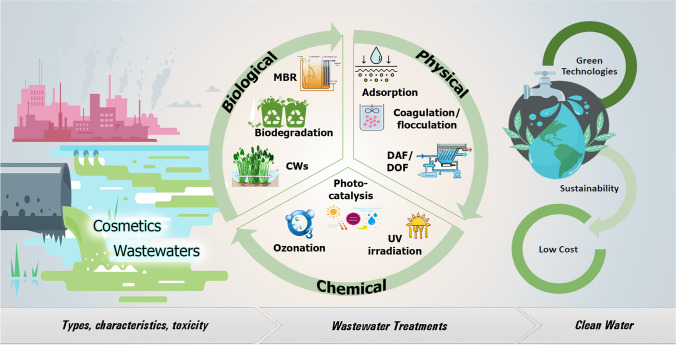
Treatment technologies of wastewaters discharged from cosmetic industries

The wastewater is characterized by attributes such as total suspended or dissolved solids, pH level, organic load, chemical or biochemical oxygen demand, and active ingredients (Yenkie 2019). The treatments are most impactful when conducted in stages, including pre-treatment and various sludge treatment methods (Crini and Lichtfouse 2019). A variety of processes have been employed to degrade or extract cosmetics from the environment (Fig. 1).

New emerging contaminants are being continuously developed, adding to the pool of cosmetic pollutants at a growing pace. A list of representative analytes (Table 1) representing a variety of cosmetics has been identified for this study.

**Table 1:** List of cosmetics identified

Category	Group	Subgroups	Compound	Chemical name	CAS number
Cosmetics	Pharmaceutical contaminants	Estrogens and hormones	Estradiol	(8R,9S,13S,14S,17S)-13-methyl-6,7,8,9,11,12,14,15,16,17-decahydrocyclopenta[a]phenanthrene-3,17-diol	50–28-2
	Pharmaceutical contaminants	Estrogens and hormones	Ethinylestradiol	(8R,9S,13S,14S,17R)-17-ethynyl-13-methyl-7,8,9,11,12,14,15,16-octahydro-6H-cyclopenta[a]phenanthrene-3,17-diol	57–63-6
	Pharmaceutical contaminants	Estrogens and hormones	Estriol	(8R,9S,13S,14S,16R,17R)-13-methyl-6,7,8,9,11,12,14,15,16,17-decahydrocyclopenta[a]phenanthrene-3,16,17-triol	50–27-1
	Pharmaceutical contaminants	NSAIDS	Diclofenac	2-[2-(2,6-dichloroanilino)phenyl]acetic acid	15,307–86-5
	Pharmaceutical contaminants	NSAIDS	Ibuprofen	2-[4-(2-methylpropyl)phenyl]propanoic acid	15,687–27-1
	Pharmaceutical contaminants	Alkylphenols	Bisphenol A	2,2-Bis(4-hydroxyphenyl)propane	80–05-7
	Pharmaceutical contaminants	NSAIDS	Cholesterol	(3S,8S,9S,10R,13R,14S,17R)-10,13-dimethyl-17-[(2R)-6-methylheptan-2-yl]-2,3,4,7,8,9,11,12,14,15,16,17-dodecahydro-1H-cyclopenta[a]phenanthren-3-ol	57–88-5
	Cosmetic ingredients	(UV) filter	Ultraviolet (UV) filter benzophenone-3 (BP-3)	2-hydroxy-4-methoxybenzophenone	131–57-7
	Cosmetic ingredients	(UV) filter	Benzophenone	Benzophenone/diphenylmethanone	119–61-9
	Cosmetic and pharmaceutical preservatives	Parabens	Methylparaben (MP)	Methyl 4-hydroxybenzoate	99–76-3
	Cosmetic and pharmaceutical preservatives	Parabens	Ethylparaben (EP)	Ethyl 4-hydroxybenzoate	120–47-8
	Cosmetic and pharmaceutical preservatives	Parabens	propylparaben (PP)	Propyl 4-hydroxybenzoate	94–13-3
	Cosmetic and pharmaceutical preservatives	Parabens	Butylparaben (BP)	Butyl 4-hydroxybenzoate	94–26-8
	PPCPs	Corrosion inhibitors	Benzotriazole, 1,2,3-benzotriazole itself (BTri)	2H-benzotriazole	95–14-7
	PPCPs	Corrosion inhibitors	Benzothiazole-2-sulfonate (BTSA)	Benzothiazole-2-sulfonic acid	941–57-1

### Physical treatment technologies

Physical treatments use physical effects without altering the composition of the wastewater. The wastewater does not affect the chemical characteristics of the contaminants, but only isolates the contaminants from water. Such approaches include the use of natural forces (gravity, van der Waals forces, etc.) and physical barriers to extract the pollutants. Sedimentation, membranes, electro-dialysis, and ion exchange are prime examples of physical treatments (Li 2020). Table 2 examines the advantages and constraints thereof.

**Table 2:** Advantages and limitations of the physical treatment options

Treatment technology	Advantages	Limitations
Adsorption	Technologically simple (simple equipment) and easy to accommodate for multiple formats Targets multiple pollutants Very efficient process with fast kinetics Outstanding quality of the treated effluent (Crini and Lichtfouse 2019) Activated carbon treatments are deemed as financially viable options and are already used in some treatment facilities (Hadla et al. 2016; Rout et al. 2021)	The adsorption effectiveness relies on the types of the contaminants, the properties of the adsorbent, as well as other environmental circumstances (Luo et al. 2014; Rout et al. 2021) Destruction of the adsorbent (might need to be incinerated, regenerated or replaced) Regeneration is costly and leads to loss of material (Crini and Lichtfouse 2019)
Coagulation and flocculation	Simple process Integrated physicochemical process A variety of chemicals is already commercially produced Low capital requirements Acceptable sludge settling and dewatering results Notable decrease in the chemical and biochemical oxygen demands (Crini and Lichtfouse 2019)	Adjunction of non-reusable materials necessary Requires the monitoring of the PH levels of the effluent Results in higher sludge amounts, which require management, treatment, and further expenses Ineffective in the extraction of arsenic (Crini and Lichtfouse 2019)
Dissolved air flotation	The solid design, brief retention time, high hydraulic loads, and small size of flocculation and flotation chambers, which allow for low capital costs (Rybachuk and Jodłowski 2019)	There are concerns regarding the mechanism of bubble/particle (aggregates) interactions besides the adhesion via hydrophobic forces (Rubio et al. 2002)

#### Adsorption technology

**Fig. 2:**
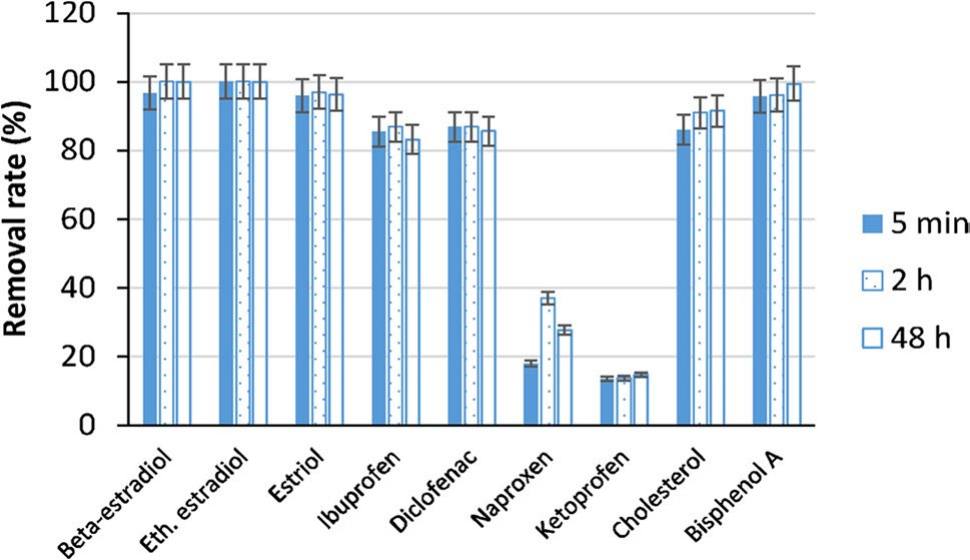
Elimination rate of 9 evaluated micropollutants from spiked wastewater after 5 min, 2 h, and 48 h BCDP treatment (Fenyvesi et al. 2020)

Adsorption has been extensively employed due to its effectiveness and simple working conditions (Gorzin and Bahri Rasht Abadi 2018) or the elimination of PCPs from the environment (Wang et al. 2017). In an effort to improve the adsorption ability, different adsorbents have been tested for the adsorption pollutants from water. Figure 2 demonstrates the rapid nature of the sorption process, achieving the removal rate of equilibrium levels for the evaluated foulants in only 5 min for most cases, after two regeneration cycles. A small improvement in time was noted for some substances, for example, naproxen and cholesterol, after a total of 2 h, and an even smaller one after 48 h (Fenyvesi et al. 2020).

Graphene oxide can be created through the oxidation of graphite (Wang and Wang 2016). Graphene and graphene oxide (GO) may be used for the extraction of PPCPs, whose removal efficiency relies on the characteristics of the adsorbate. Similarly to the activated carbon, pH level and contact duration have an evident impact on the extraction efficacy of graphene and its oxide (Kyzas et al. 2015; Yang and Tang 2016). They both have higher specific surface area than AC, which improves their potential to remove the PPCPs (Wang and Wang 2016). Mehreen Iqbal et al. described the single-step preparation of an rGO/Ag_2_O nanocompound for wastewater treatment, which they later characterized using various methods. More specifically, XRD data verified the synthesis of the nanocomposite. The SEM analysis revealed that the carbon sheet was randomly connected with Ag_2_O, while the average particle size was estimated to be approximately 25 nm. The nanocomposite exhibited efficient catalytic reduction of 4-nitrophenol and an outstanding degradation of methyl blue and brilliant green (Iqbal et al. 2021).

Similarly, carbon nanotubes possess desirable properties, rendering them ideal options for various applications. Multiple research works have examined the elimination of substances such as ketoprofen (Liu et al. 2014a; Liu et al. 2014b), carbamazepine (Liu et al. 2014a; Liu et al. 2014b), sulfamethoxazole (Ji et al. 2009), and triclosan using carbon nanotubes (Cho et al. 2011). The results suggest that carbon nanotubes have high adsorption ability versus the PPCPs, which did depend on the surface chemistry and characteristics of CNTs. In addition, the attributes of the PPCPs may affect the adsorption process. Further details about the elimination of PPCPs by CNTs can be found in the past review (Jung et al. 2015), which examined the elimination of PPCPs by CNTs.

Wang and Chu (2016) reported that MTCNTs are effective in the elimination of substances such as triclosan, ibuprofen, and caffeine and the extraction efficiency of PPCPs improved while the feeding concentration decreased. Furthermore, they noted that a larger inner diameter did not improve the adsorption and the performance versus the competing fulvic acid existing in the PPCP-contaminated water.

#### Coagulation and flocculation

Flocculation is an encouraging, inexpensive technique that may act as the initial step in the dewatering and harvesting processes. It is often referred to as coagulation, even though their definition is not the same. More specifically, coagulation revolves around the adjustment of the pH levels and introduction of an electrolyte, while flocculation relies on the cationic addition of polymers. Nevertheless, both have been found to perform in the same manner (Jeevanandam et al. 2020). Coagulants can be introduced to the wastewater in an effort to force the smaller particles to aggregate into larger ones that can be later settled (Amuda and Alade 2006). The process is based on the neutralization of colloids with negative charge, through the cationic hydrolysis and by incorporating the pollutants in hydroxide (Duan and Gregory 2003). One of the main attributes of coagulation is the extraction of organic materials and suspended solids (Amuda and Alade 2006). Common coagulants include aluminum, iron salts, and lime. The aggregated contaminants may later be extracted via sedimentation or floatation (Plattes et al. 2007).

The improvement of the efficiency of the coagulation-flocculation flow has been widely explored in the past. Partial polymerization appears to be the best option for the enhancement of simple A1 salts, upon analyzing their aquatic chemistry and behavior, which resulted in the production of a variety of pre-polymerized aluminum solutions (Sohrab and International Association of Mechanical Engineers, World Scientific and Engineering Academy and Society 2008).Over the past 20 years, the most popular pre-polymerized coagulants compose of poly-aluminum chloride, poly-sulfate, and poly-chloro-sulfate (PAC, PAS, PACS, respectively) (“Copperas as Iron-Based Coagulant for Water and Wastewater Treatment,” 2021). As a consequence, polymeric aluminum and/or iron composites, such as PFSiS (polyferric silicate sulfate), PASiC (poly-aluminum silicate chloride), PSiFAC (poly-aluminum ferric silicate chloride), and PSiFAC-Mg (poly-aluminum ferric silicate magnesium chloride), have been studied chiefly in a laboratory setting, in regard to their ability to remove turbidity and arsenic, treat high-strength industrial wastewaters, and reduce fouling in membrane bio-reactor systems (Zouboulis and Moussas 2008).

In 2019, Tolkou et al. compared the newly introduced coagulant PSiFAC-Mg30-10–15 to PSiFAC-Na1.5–10-15, which is another pre-polymerized, Al-based coagulant, with no magnesium however, that has already been explored in terms of lowering the fouling levels in membrane bioreactor systems and the removal of arsenic. Furthermore, the comparison was extended to the typically used and commercially available AlCl3 in regard to the removal of fluoride from simulated polluted groundwaters. The outcome suggested that PSiFAC-Mg30-10–15 was more effective than the materials with no magnesium. The residual aluminum quantities in the treated wastewaters were studied for all coagulants under various pH levels and while considering a variety of coagulant concentrations below the maximum limit of Al in potable water (200 mg/L). It should be noted that a minor increase of magnesium concentration in the treated water can be counted as an additional asset of the novel coagulant (Tolkou et al. 2019).

In another study, Tolkou et al. reported that the PSiFAC1.5:10:15 coagulant is more efficient in the C/F process, specifically in terms of COD removal, regardless of if a flocculant aid (polyelectrolyte) is used. The remaining aluminum concentration was studied and found to be lower than the maximum limit. Further cost benefits may emerge by the use of this material in particular wastewater treatment scenarios, including the potential lack of requirement for equipment to handle the organic polyelectrolyte reagent. As a result, the treatment process becomes simpler, and the total cost can be reduced (Tolkou and Zouboulis 2020).

**Fig. 3:**
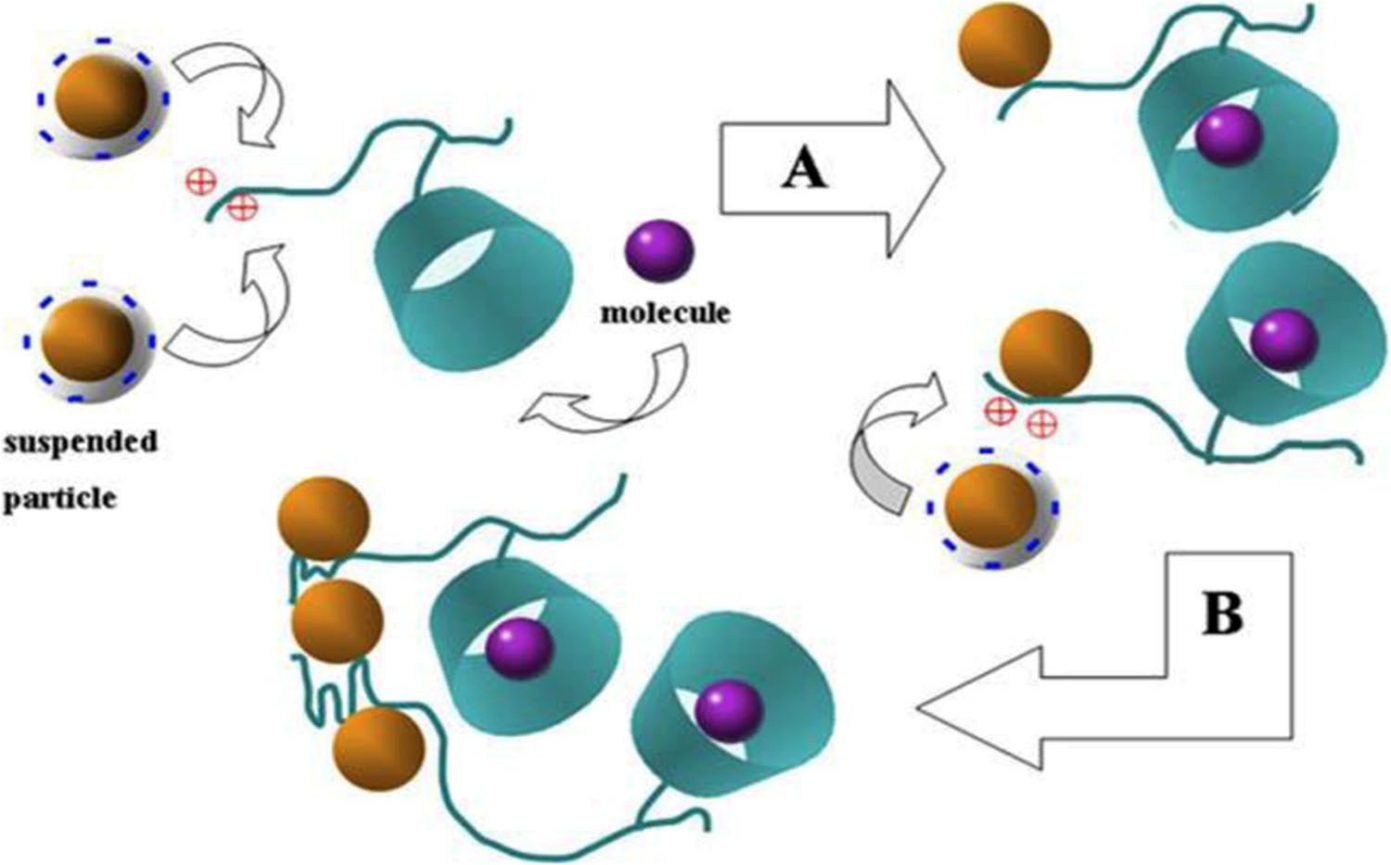
The flocculation mechanism of cationic polyacrylamide (Tang et al. 2020)

A depiction of the stages of the flocculation mechanism is presented in Fig. 3. Initially, the polymers adsorb particles and molecules using the electrical neutralization and the inclusion effect of the β-CD cavity. The other segment of the particles can later be adsorbed by a different polymer chain, thus formulating bridges, and causing the particles to convene into clumps (Tang et al. 2020).

Studies on host compounds have verified that the structure of a cyclodextrins (CD) greatly contributes to the effectiveness of its inclusion effect and discussed the inclusion function of β-CD into an acrylamide polymer. Furthermore, they studied the flocculence acrylamide/allyl-β-cyclodextrin/dimethyl diallyl ammonium chloride, otherwise referred to as CDM-16, and explored its flocculation mechanism. The authors examined the flocculation effects of acrylamide polymers, comparing those with or without the β-CD side groups and alleged that the version with the side-groups was more effective, attributing this to the inclusion function of β-CD, as well as the positively charged dimethyl diallyl ammonium chloride (DMDAAC) that has the ability to adsorb negative particles via electric neutralization (Tang et al. 2020).

#### Dissolved air flotation

Flotation is a separation process, where gas bubbles are utilized as the means of transportation. Suspended particles, which are either hydrophobic or rendered to be so, are then attached to these bubbles and float toward the surface of the solution, against the direction of gravity. There are various bubble generation mechanisms, broadly categorized as dispersed-air flotation, which often includes electroflotation, or as dissolved-air flotation, which is based on Henry’s law (Kyzas and Matis 2018).

#### Cosmetics removal by physical methods

Table 3 summarizes the significant findings of various physical treatments categorized according to the mechanisms employed to remove cosmetics, antibiotics, hormones, biocides, and PPCPs. Adsorption was found to be the main mechanism responsible for the extraction of the examined materials considering the high removal efficiency and the short period of time required to achieve these results.

**Table 3:** Removal of cosmetics from wastewaters by physical methods categorized according to their mechanism

Compound	Material	Initial concentration	Removal (%, time)	Mechanism	Ref
Turbidity (TUR), suspended solids, (SS), and chemical oxygen demand (COD)	Cactus tree, species *Opuntia ficus-indica* as flocculant, Aluminum sulfate (AS) as coagulant and fresh cladodes juice (FCJ) as bioflocculant	0.5 g/L AS 5 mL FCJ	TUR 93.65%, SS 82.75% COD and 64.30% after 30 min	Coagulation flocculation and sedimentation	(Rachdi et al. 2017)
4‑nitrophenol	rGO/Ag_2_O nanocomposite	3 mL of 0.1 mM 4-NP solution	97% after 35 min	Adsorption	(Iqbal et al. 2021)
Estradiol, ethinyl estradiol, estriol, diclofenac, ibuprofen, bisphenol A, and cholesterol	Cyclodextrin bead polymer	1 kg activated BCDP treated 300 L of effluent. Poured through columns with 40.8 L volume	Between 85 and 99% depending on the compound, after 5 min	Adsorption	(Fenyvesi et al. 2020)
Chemical oxygen demand (COD), Total suspended solids (TSS)	A1 6010	1 mL/L	COD 78.8%, TSS 95.2% After 10 min	Coagulation and Dissolved air flotation	(Wiliński et al. 2017)
Chemical oxygen demand (COD), Total suspended solids (TSS)	A1 6010	1 mL/L	COD 79.1%, TSS 94.4% After 10 min	Coagulation and Dissolved ozone flotation	(Wiliński et al. 2017)
Chemical oxygen demand (COD), Total suspended solids (TSS), Various micropollutants	A1 3010	1 mL/L	COD 81.3%, TSS 96.3%, VMP 93.8% After 10 min	Coagulation and Dissolved air flotation	(Wiliński et al. 2017)
Chemical oxygen demand (COD), Total suspended solids (TSS), Various micropollutants	A1 3010	1 mL/L	COD 81.1%, TSS 96.3%, VMP 96.3% After 10 min	Coagulation and Dissolved ozone flotation	(Wiliński et al. 2017)
Sample 5, Chemical oxygen demand (COD)	Al_2_(SO_4_)_3_	125 mg/L	COD 68% after 2 min	Dissolved air flotation	(Bogacki et al. 2017)
Sample 5, Chemical oxygen demand (COD)	Al 3010 Al	1 mg/L	COD 68% after 2 min	Dissolved air flotation	(Bogacki et al. 2017)
Sample 5, Chemical oxygen demand (COD)	Al 3010 Al	1 mg/L	COD 77% after 2 min	Dissolved air flotation	(Bogacki et al. 2017)
Sample 3, Chemical oxygen demand (COD)	Al_2_(SO_4_)_3_	125 mg/L	COD 77.1% after 2 min	Dissolved air flotation	(Bogacki et al. 2017)
Sample 3, Chemical oxygen demand (COD)	Al 3010 Al	0.5 mg/L	COD 72.9% after 2 min	Dissolved air flotation	(Bogacki et al. 2017)
Sample 3, Chemical oxygen demand (COD)	Al 3010 Al	0.5 mg/L	COD 75.6% after 2 min	Dissolved air flotation	(Bogacki et al. 2017)

Rachdi et al. (2017) suggested that a treatment using aluminum sulfate as a coagulant along with cactus juice (*Opuntia ficus-indica* species) as a natural flocculant, resulted in considerably better removal efficiency of turbidity (~ 94%), chemical oxygen demand (~ 64%), and suspended solids (~ 83%) in the treated water.

Iqbal et al. (2021) used adsorption for the removal of 4-nitrophenol (4-NP) using a graphene oxide silver oxide (rGO/Ag_2_O) nanocomposite. The process involved the reduction of 4-NP into 4-aminophenol (4-AP), allowing nanocomposite to be efficiently reused for a minimum of five additional cycles without any noticeable loss.

Fenyvesi et al. (2020) evaluated the capacity of epichlorohydrin-crosslinked *β*-cyclodextrin polymer (BCDP) sorption technology to remove some of the most typical contaminants, concluding that it can successfully extract substances such as estradiol, diclofenac, ibuprofen, and bisphenol with an efficiency that ranges between 85 and 99%. BCDP however cannot be regenerated.

Wiliński et al. (2017) explored the pretreatment of cosmetic wastewater through dissolved air and ozone flotation (DAF and DOF). Various different coagulants were selected for the tests, such as Al 3010 and 6010, PAX XL19, Flokor 1S, and Megafloc, out of which, the ones with the highest efficiency were chosen, i.e., Al 3010 and Al 6010. The findings revealed that both approaches exhibit similar chemical oxygen demand removal, reaching about 79%, while total suspended solids removal reaches about 95% and 94%, respectively. The assessment of the results verified that the DOF process using Al 3010 performed better than DAF. Total suspended solids were also removed at a 96.3% rate, while 81.3% chemical oxygen demand removal was documented.

Wastewater containing samples of various types of cosmetics were treated using dissolved air flotation assisted by coagulation. Bogacki et al. (2017) demonstrated that the effectiveness of the process depended on the coagulant used in the treatment process and the type of sample. The highest chemical oxygen demand removal rate was achieved for sample 5 (shampoos and lotions), using Al_2_(SO_4_) as the coagulant. The authors also verified that this method is ineffective towards sun screen UV filters (sample 4). The raw wastewater chemical oxygen demand ranged between approximately 285 and 2125 mg/L, and the effectiveness of the processes relied on the various coagulants and industrial profile. The removal of chemical oxygen demand ranged between 11 and 78%.

### Biological treatment technologies

Biological wastewater treatment approaches are applicable to carbonaceous organic materials, representing — among others — the removal of BOD and phosphorus, or the nitrification and denitrification. Biological processes may be categorized as aerobic or anaerobic. The aerobic ones often tend to achieve better results, while anaerobic bacteria use the notions of resource recovery and utilization to limit the pollution (Li 2020). Table 4 describes the advantages and limitations of such approaches.

**Table 4:** Advantages and limitations of the biological treatment technologies

Treatment technology	Advantages	Limitations
Aerobic-anaerobic	Increased purification levels, ability to manage high organic loads, generation of limited amounts of sludges that are often quite stable, and production of methane as end-product (Aziz and Abu Amr 2019)	Anaerobic treatment requires time (Samer 2015)
Activated sludge	Inexpensive (Onesios et al. 2009)	Incomplete degradation leading to the creation of toxic degradation by-products Non-effective in the removal of recalcitrant contaminants (Oulton et al. 2010), while biodegradation is affected by structural characteristics and environmental conditions (Rajasulochana and Preethy 2016) Depends on energy (Sharma and Sanghi 2012) Low availability or lack of degraders (Wang and Wang 2016)
Biodegradation	Prime method for the elimination of PPCPs (Wang and Wang 2016)	
Constructed wetlands	Low energy requirements Low operational cost (Kaur et al. 2019)	Large area footprint Required operating cost (Kaur et al. 2019)
Membrane bioreactor process	Applicable versus many contaminants (Weiss and Reemtsma 2008)	Inability to remove recalcitrant contaminants (Kaur et al. 2019)

#### Aerobic and anaerobic processes

In aerobic treatment methods, the oxygen that is dissolved in the water is used by bacteria for the degradation of organic contaminants under aerobic conditions. The related reaction is expressed by Eq. (1):

1 $$\mathrm{Organic}\;\mathrm{Matter}+\mathrm{Bacteria}+{\mathrm O}_2\rightarrow\mathrm{Bacteria}+{\mathrm H}_2\mathrm O+{\mathrm{CO}}_2+\mathrm{By}-\mathrm{products}$$

It is obvious that oxygen is crucial for the conversion; thus, air should be consistently supplied inside the tank. Other affecting factors include time, temperature levels, bacteria characteristics, and pH levels. This method is useful for the elimination of volatile, dissolved, or suspended organics, phosphates, biological and chemical oxygen demand, nitrates, etc. It allows for approximately 90% less organic waste; however, the excess bio-solids will need further treatment technologies that are relatively expensive (Bolisetty et al. 2019; Gupta et al. 2012).

The anaerobic process occurs when there is a deficit of oxygen. This process requires the use of bacteria to degrade waste into nontoxic by-products, releasing gases such as methane and nitrogen. Equation (2) depicts the reaction mechanism for anaerobic processes:

2 $$\mathrm{Organic}\;\mathrm{Matter}+\mathrm{Bacteria}\rightarrow\mathrm{Bacteria}+{\mathrm{CO}}_2+{\mathrm{CH}}_4+\mathrm{By}-\mathrm{products}$$

Anaerobic methods can handle wastewater with chemical oxygen demand values exceeding 4 g/L, as opposed to aerobic processes that may only treat wastewater with chemical oxygen demand values of up to 1 g/L. The primary advantage of anaerobic treatment is that it requires less energy and allows for the extraction of beneficial nutrients. The operating conditions that should be monitored while using an anaerobic system are the temperature levels and toxicity. The most important drawback of anaerobic wastewater treatment is that it requires a relatively long time to complete (Bolisetty et al. 2019; Chan et al. 2009; Gupta et al. 2012).

Aerobic and anaerobic biodegradations have different effects depending on the type of PPCPs. For instance, diclofenac may be effectively eliminated through anaerobic biodegradation, while anti-inflammatory drugs such as ibuprofen and naproxen as well as lipid regulators require aerobic biodegradation (Huang et al. 2011).

#### Activated sludge process

This approach requires a lower cost as opposed to other more complex options. Due to the PPCPs’ low concentration levels, which are not high enough to sustain the development of microorganisms, catabolism plays a major role in biodegradation (Onesios et al. 2009). The effectiveness of the treatment is affected by operational factors, such as hydraulic and sludge retention times (HRT & SRT). Longer HRTs improve the elimination rates for most PPCPs (Vergili 2013). The drawback of this mechanism lies in its inability to extract recalcitrant PPCPs (Oulton et al. 2010).

**Fig. 4:**
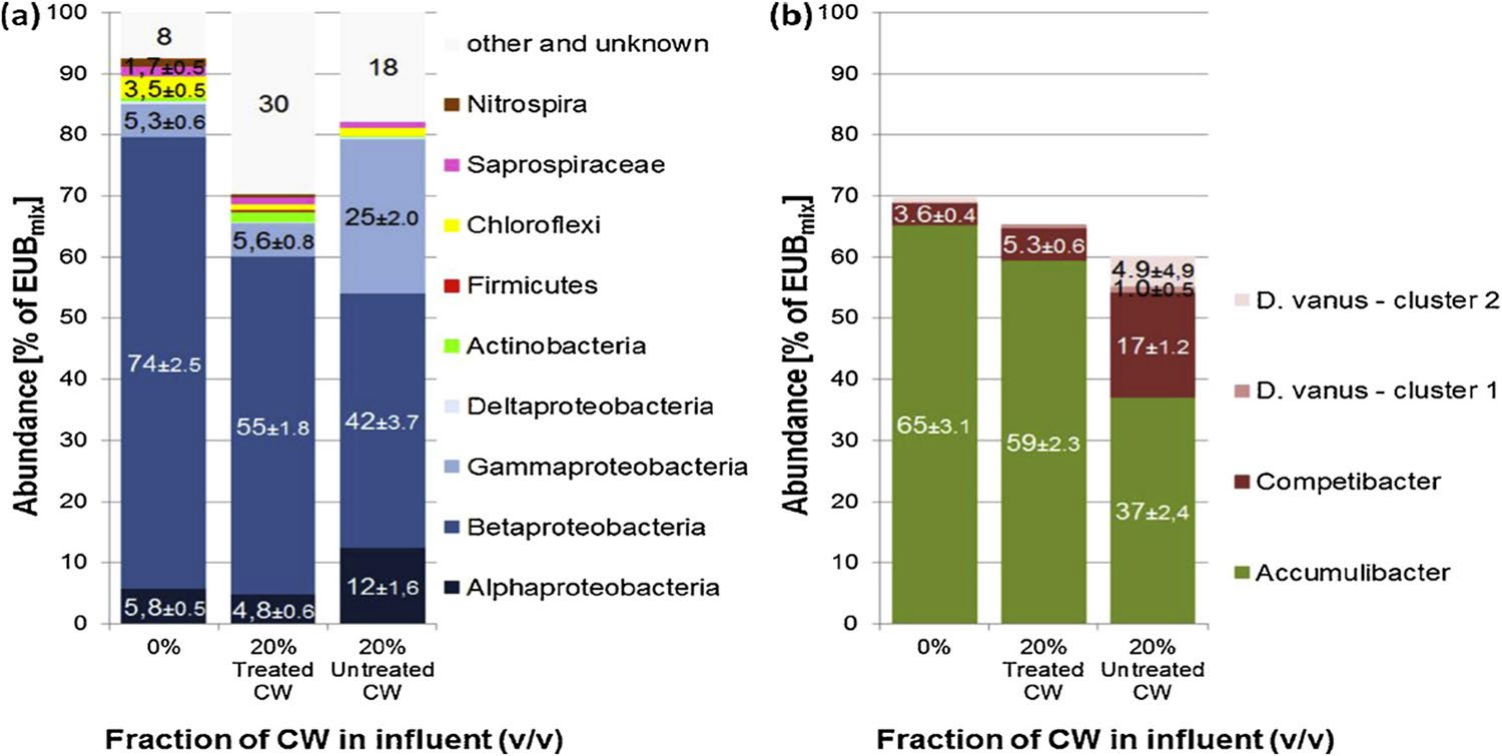
Structure of the microbial community (**a**) and abundance of polyphosphate and glycogen accumulating organisms (PAOs and GAOs) (**b**) in the AS of the SBR (Muszyński et al. 2019)

The lowered efficiency in the sequencing batch reactor (SBR) was paired with alterations in the composition of the sludge (Fig. 4). Both the treated and untreated constructed wetland (CW) resulted in lowered levels of bacteria, dropping from about 74 to 55% for the treated CW and to 42% of the bacterial biovolume for the untreated CW (Muszyński et al. 2019).

**Fig. 5:**
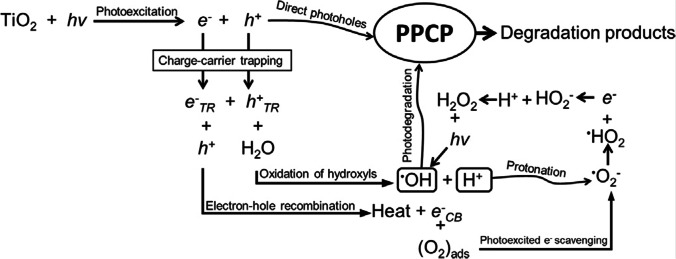
The reactions taking place during photocatalytic oxidation and reduction (Awfa et al. 2018)

The lab-scale sequencing batch reactor was used for the enrichment of the polyphosphate accumulating organisms (PAOs); however, raising the contribution of the CW in the feed progressively lowered the abundance of bacteria from a starting 65 to 59% for the treated CW and 37% of all bacteria for untreated CW (Fig. 5). A noticeable decline was recorded when untreated CW was added (Fig. 4b) (Muszyński et al. 2019).

José Luis Malvar assessed seven pharmaceuticals and personal care products, as well as their main metabolites, monitoring them using various stabilization methods, lagooning, composting, and dehydration. The evaluated samples were assessed for sixteen compounds, and it was noted that their distribution was similar in primary sludge, despite the diverse geographic locations of the wastewater treatment facilities, in accordance with the metabolic ratios of the majority of the examined compounds. Each compound exhibited different behaviors in terms of stability. Some persisted in all the reviewed technologies, whereas others were highly degradable (Luis Malvar et al. 2020). Various researchers (Kahl et al. 2017; Larsson et al. 2007; Nivala et al. 2019) have assessed the elimination rates of diclofenac in wastewater, under both aerobic and anaerobic environment, reaching rates up to almost 100% in certain cases (Larsson et al. 2014).

The results stemming from Lose Luis Malvar’s work support that adsorption is the most important elimination mechanism, as previously suggested by other researchers (Yan et al. 2019). The elevated persistence of such compounds to the biodegradation processes could be attributed to the presence of chlorine atoms, since they offer them high stability. The detection frequency was higher in primary sludge. Similar outcomes have been documented for pharmaceutical compounds (Martín et al. 2015) and personal care products (Wu et al. 2017). Advanced oxidation (Mohapatra et al. 2014), hydrothermal carbonization (vom Eyser et al. 2016), and adsorption (Malvar et al. 2020) have exhibited encouraging results on the elimination of some of the examined substances.

#### Membrane bioreactor process

Membrane bioreactor (MBR) processes promise efficient wastewater treatment and symbolize an improvement over the traditional activated sludge processes. They incorporate biological degradation and membrane filtration in a simultaneous, combined process, and offer more flexibility in terms of potential modifications required to modulate the biological performance (Tay et al. 2007). The biological part of the process transforms the dissolved organic matter into suspended biomass, decreasing the membrane fouling, thus improving the recovery rate. The membranes added in the bioreactors create an effective barrier that keeps solids and bacteria in the process tank. This technology has multiple benefits such as high quality of the resulting treated water, low sludge formulation, and small footprint and is stable and easily expandable. They are thus exceedingly suitable for the treatment of recalcitrant wastewater, where long sludge retention times are employed, enabling the effective elimination of slowly biodegradable contaminants. Membrane fouling is notably a significant disadvantage of this technology, since the need for more energy for backwashing renders it less efficient (Friha et al. 2014). An MBR system effectively regulates the sludge retention time, thus leading to a low sludge creation and to an increase in overall efficiency. It is thus anticipated that such a mechanism would effectively eliminate PPCPs.

Oulton et al. (2010) claimed that MBR is more effective for the extraction of PPCPs than the traditional activated sludge process. In addition, MBR has been reported to be effective versus PPCPs when paired with membrane processes. Mei Chen et al. introduced an innovative electrochemical membrane bioreactor (EMBR) for improving the PPCP elimination from actual municipal wastewater. EMBR displayed better results than the control MBR for 14 PPCPs, such as certain fluoroquinolones, macrolides, sulfonamides, and anti-inflammatory drugs, while no noticeable changes were documented for the remaining PPCPs. The improved results for the 14 PPCPs were mainly ascribed to electrooxidation. Furthermore, the membrane fouling rates of EMBR were considerably reduced as opposed to the control MBR system. An assessment of the microbial activity verified that the applied electric field had no significant adverse impact on microbial viability and diversity. These findings verified that this combination of contaminant elimination and membrane fouling mitigation has a strong potential to be used for the elimination of PPCPs from wastewater (Chen et al. 2020).

#### Biodegradation

Biodegradation processes take advantage of microorganisms found in natural ecosystems in order to degrade specific low-concentration organic foulants, resulting in their elimination. Biodegradation of BP-3 using sludge has already been studied (Liu et al. 2012) with Badia-Fabregat et al. (2012) suggesting that the *Trametes versicolor* fungus exhibited promising results for the biodegradation of BP-3 (Rodriguez et al. 2016). Combinations with methanol for the improvement of the biodegradation of coal-gasification wastewater have been explored in past research works. The addition of methanol has been proven to mitigate the toxicity of coal gasification wastewater and improve the degradation effectiveness (Wang et al. 2010).

#### Constructed wetlands

Constructed wetlands are an environmentally friendly, inexpensiuve technology that has proceeded to become one of the most frequently used biological treatment option. As a result, they have a promising potential for the elimination of PPCPs (Ávila and García 2015).

Matamoros and Bayona (2006) and Matamoros et al. (2010) have thoroughly explored the wetland systems for the extraction of PPCPs from wastewater, such as ibuprofen, which is the most frequently referenced. The contaminant elimination rate by CW may be impacted by seasonal variations, achieving better results during summer as opposed to winter (Hijosa-Valsero et al. 2011). The CW systems are considered largely ineffective towards recalcitrant compounds. Tejeda et al. (2017), however, sought to examine the elimination of carbamazepine using a hybrid CW system and achieved a 60% removal. As a result, CW systems are deemed capable of achieving high removal rates of PPCPs, under optimal conditions, which would establish them as a sustainable option of wastewater treatment.

Huma Ilyas et al. examined the effectiveness of four types of constructed wetlands: free water surface, horizontal or vertical flow, and hybrid CWs for the elimination of 20 personal care products (PCPs), according to secondary data stemming from 39 reviewed papers on 137 types of CWs. Despite the significant variation in the elimination rate of PCPs, CWs have been proven to be an effective treatment technology. The removal efficiency of fifteen frequently studied materials ranged between 9 and 84%. Even though CWs mitigated the environmental risks brought on by various PCPs, triclosan is still categorized as high risk due to its effluent concentration. Five other PCPs were deemed to be of medium risk (such as triclocarban and methylparaben). In most cases, adsorption is the most frequently used extraction mechanism. Hybrid CWs were relatively more efficient, possibly due to the co-existence of aerobic and anaerobic conditions, and the longer hydraulic retention time boosting the elimination rate of PCPs (Ilyas and van Hullebusch 2020).

#### Cosmetics removal by biological methods

Similar to the physical removal methods, the various biological methods for the extraction of substances, such as cosmetics, antibiotics, hormones, biocides, and PPCPs, have been summarized in Table 5. The membrane bioreactor process was found to be the main removal mechanism for the elimination of the majority of the studied materials. Constructed wetlands and biodegradation are additional mechanism that are frequently encountered.

**Table 5:** Removal of cosmetics by biological methods

Compound	Material	Initial concentration	Removal (%, time)	Mechanism	Ref
Ultraviolet (UV) filter benzophenone-3 (BP-3) in oxic and anoxic conditions (nitrate, sulfate, and Fe [III]-reducing)	10% activated sludge	1 mg/L	84.7–94.1% after 42 days	Aerobic and anaerobic processes	Liu et al. (2012)
DOC	MBR -sludge	5 g/L	79% after 4 w	Membrane bioreactor process	Weiss and Reemtsma (2008)
BTri	MBR-sludge	5 g/L	61% after 4 w	Membrane bioreactor process	Weiss and Reemtsma (2008)
5-TTri	MBR-sludge	5 g/L	61% after 4 w	Membrane bioreactor process	Weiss and Reemtsma (2008)
BTSA	MBR-sludge	5 g/L	65 ± 16% after 4 w	Membrane bioreactor process	Weiss and Reemtsma (2008)
2-NSA	MBR-sludge	5 g/L	94 ± 4% after 4 w	Membrane bioreactor process	Weiss and Reemtsma (2008)
1-NSA	MBR-sludge	5 g/L	92 ± 4% after 4 w	Membrane bioreactor process	Weiss and Reemtsma (2008)
Benzophenone-3 (BP-3)	*Methylophilus* sp. strain FP-6	5 mg/L	65% after 8 d	Biodegradation	Jin et al. (2019)
Biocides, steroid hormones, antibiotics, PPCPs	Tidal flow constructed wetlands (TFCWs) with baffle	-	B 92.4%, SH 99.5% A 77.2%, PPCPs 92.9% after 24 h	Constructed wetlands	Cheng et al. (2021)
Biocides, steroid hormones, antibiotics, PPCPs	Tidal flow constructed wetlands (TFCWs) with plants	-	B 93.4%, SH 98.5% A 85.2%, PPCPs 94.3% after 24 h	Constructed wetlands	Cheng et al. (2021)
Biocides, steroid hormones, antibiotics, PPCPs	Tidal flow constructed wetlands (TFCWs) with both baffle and plants	-	B 97.1%, SH 99.8% A 90.2%, PPCPs 97.4% after 24 h	Constructed wetlands	Cheng et al. (2021)

The removal of various contaminants from municipal wastewater using a lab-scale MBR system was evaluated by Weiss and Reemtsma et al. (2008). Their findings indicate that for half of the examined materials, one-step MBR treatment was evidently superior to traditional activated sludge treatment with anaerobic and aerobic stages. For those substances (Btrio, DOC, 5-TTri, etc.), the removal rate ranges between 61 and 94%. Furthermore, the process resulted in lower effluent concentrations, ranging between 22 and 56%. A hydraulic retention time (HRT) of 7 h appears to be enough for the extraction of trace contaminants.

Liu et al. suggested that anaerobic degradation of BP-3, which requires a half-time of about 3 days, is more effective than aerobic degradation which has a half time of about 10 days (Liu et al. 2012).

Jin et al. (2019) demonstrated that, under ideal conditions, the degradation rate of benzophenone-3 (BP-3) may reach approximately 65% after 8 days when using *Methylophilus* sp. strain FP-6.

Jin et al.’s (2019) work reported that a Gram-negative aerobic bacterium that has the capacity to degrade benzophenone-3 as a single carbon source was retrieved from a municipal wastewater treatment facility and was categorized as *Methylophilus* sp. FP-6. Methanol was selected for additional tests as a co-metabolic carbon source to boost the microbial degradation efficacy of BP-3. Various experiments were conducted to assess the optimal degradation conditions, under which the BP-3 degradation rate reached approximately 65% after 8 days of incubation. Based on the evaluation of the detected metabolic intermediates, three different routes for the degradation of BP-3 by this strain were proposed.

Cheng et al. (2021) investigated three tidal flow constructed wetlands (TFCWs) with diverse alterations (baffle, plants, both baffle, and plants) in order to treat sewage and specially to assess the PPCP elimination efficacy and mechanism. A total of 24 PPCPs were identified in the influents. It was noted that modification with both baffle and plants considerably affected the extraction of PPCPs. They studied that modification with both baffle and plants considerably affected the extraction of PPCPs and more specifically biocides (97.1%), steroid hormones (99.8%), antibiotics (90.2%), and PPCPs (97.4%) within 24 h. According to the mass balance assessment, the microbial degradation was the main removal mechanism with a percentage reaching almost 86%, followed by substrate adsorption (about 14%) and plant uptake (less than 0.5%). Further analysis suggested that the inclusion of baffle and plants improved the elimination efficiency of PPCPs by promoting microbial diversity and altering the dominant microorganisms.

### Chemical treatment

The chemical treatment of wastewater can have various effects, such as the generation of insoluble solids and gases, the formulation of biodegradable compounds from non-biodegradable ones, and the destruction or deactivation of chelating agents that can efficiently remove substances from wastewater. In some cases, the coagulant links the colloidal particles through slow agitation. Some materials can be chemically oxidized to procure safer materials such as CO_2_ and water (Li 2020). Table 6 presents a list of potential chemical treatments as well as their advantages and limitations.

**Table 6:** Advantages and limitations of chemical methods

Treatment technology	Advantage	Limitations
Fenton	The on-site creation of H_2_O_2_, which can bypass the risks linked to its transportation, storage, and management; The continuous regeneration of Fe^2+^, which can hinder the iron sludge generation and enhance the degradation effectiveness (Zhang et al. 2019)	Low pH level requirement High sludge production Pharmaceuticals may aggregate in the iron sludge created after the treatment (Mahtab et al. 2022) Limited H_2_O_2_ yield Low unit cell body throughput. Low levels of density and conductivity (M. Zhang et al. 2019)
Ozonation	Simple, quick, and effective Generation of ozone *on*-*site* (Crini and Lichtfouse 2019) High elimination rate (Dhodapkar and Gandhi 2019) Full mineralization of microcontaminants (Kaur et al. 2019)	Short half-life (ozone) No effect on salinity (ozone) (Crini and Lichtfouse 2019)
Photocatalysis	High degradation percentage (Cheng et al. 2019)	Exposure to carcinogenic UV light (Cheng et al. 2019)

#### Advanced oxidation processes

The advanced oxidation process (AOP) has been described as an emerging mechanism for the mineralization of organic contaminants as an efficient treatment option that requires the in situ production of radicals that are able to degrade and remove organic contaminants from the environment.

AOP may effectively extract dangerous pollutants or mineralize them, because of the generation of oxidizing agents such as radicals and superoxides (Anjali and Shanthakumar 2019). It might be achieved through ozonolysis or homo-/heterogeneous catalyzed oxidation, or photocatalysis, which is one of the green technologies that has recently received the spotlight as a viable alternative for wastewater treatment due to being inexpensive, non-toxic, and effective in the elimination of foulants (Hou et al. 2020). AOPs can be categorized into:

- Photochemical processes such as UV oxidation, UV/ultrasound, photocatalysis, and microwave
- Non-photochemical processes such as ozonation, electron-beam irradiation, and wet-air oxidation (Gültekin and Ince 2007)

#### Ozonation

Ozone (O_3_) has an enhanced oxidation ability, thus is expected to oxidize organic contaminants more effectively. Using advanced oxidation technologies based on ozone is beneficial because ozone is a good oxidation agent that can handle a variety of organic foulants and is able to produce hydroxyl radicals when combined with H_2_O_2_ or UV irradiation. In addition, ozone is also frequently used in potable water treatment, due to its ability to offer microbial disinfection and oxidation of low concentration pollutants in reused wastewater (Cuerda-Correa et al. 2016).

#### Photocatalysis

Among the various advanced oxidation processes, the heterogeneous photocatalysis using semiconductor catalysts (TiO_2_, Fe_2_O_3_, GaP, etc.) has proven to be effective in degrading a variety of ambiguous refractory organics into easy to biodegrade compounds, followed up by the mineralization to carbon dioxide and water. Titanium dioxide has already received a lot of research attention, since it is a very active photocatalyst under the photon energy range between 300 and 390 nm and retains its stability after multiple catalytic cycles, whereas other materials, such as Cds or GaP, may be degraded, formulating toxic by-products in the process. Furthermore, the chemical and thermal stability and resistance to chemical breakdown have attributed to the wide adoption of titanium dioxide in photocatalytic water treatments (Chong et al. 2010).

The most important reactions in the photocatalytic oxidation and reduction mechanism are illustrated in Fig. 5 (Awfa et al. 2018).

#### Fenton and photo-Fenton processes

**Fig. 6:**
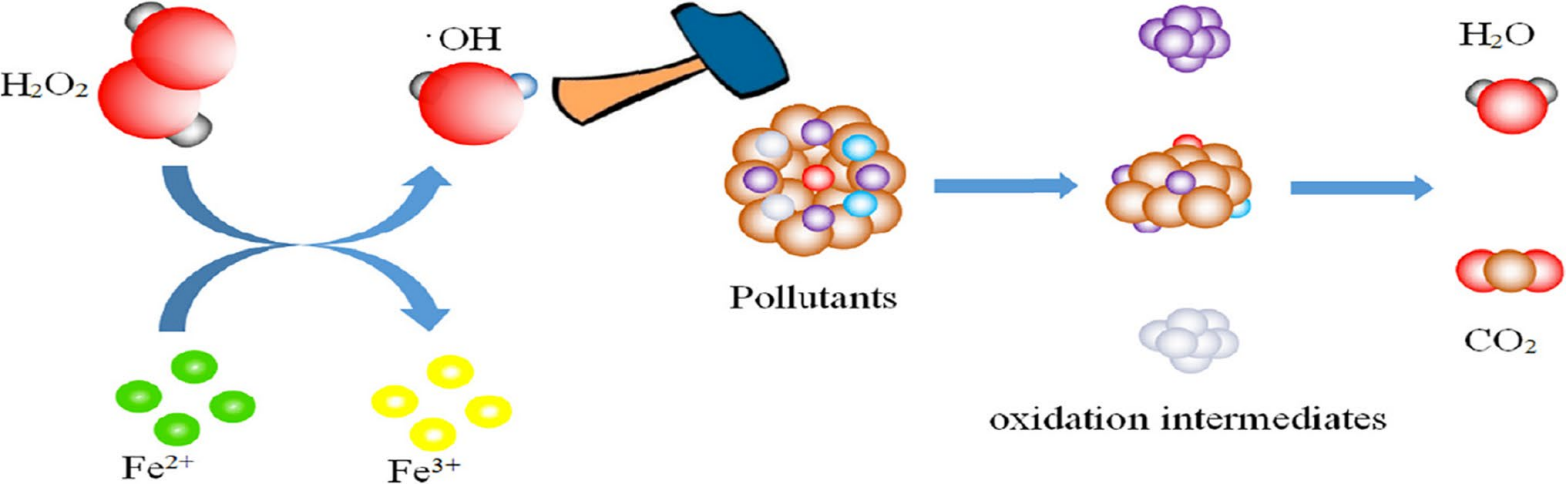
Reaction mechanism for the Fenton process (Zhang et al. 2019)

The Fenton reaction, originally discovered in 1984, is probably the oldest advanced oxidation process and is frequently referred to as the origin of advanced oxidation processes. Despite its simplicity and effectiveness, and its ability to combine with artificial or solar irradiation (photo or solar Fenton), the requirement for low PH levels, the sludge production, and the resulting iron separation from the effluent do not make it ideal for widespread industrial uses (Frontistis 2021). Fenton’s oxidation reaction uses a combination of hydrogen peroxide and Fe^2+^ which created hydroxyl radicals (OH•) in an at acidic pH levels and ambient temperature (Perdigón-Melón et al. 2010). The process involves the formulation of reactive oxidizing species, with the ability to efficiently degrade the foulants of the effluent in acidic pH levels and involves oxidation, neutralization and coagulation mechanisms (Sansebastianmartinez 2003). To get past the barriers of the classical Fenton process, the electro-Fenton process has been established (de Luna et al. 2012), while the rest of the process remains the same (Ganiyu et al. 2018). The oxidation mechanism for the Fenton process is depicted in Fig. 6 (Zhang et al. 2019).

The photo-Fenton process is an effective advance oxidation process for the treatment of MPs, which uses UV radiation to create OH^−^ radicals in the presence of iron catalysts and to destroy the pollutants efficiently (Ahmed et al. 2017). Improved production of the radicals can be achieved in acidic or almost neutral pH conditions. In these pH ranges, Fe^3+^ cations formulate various light absorptive hydroxyl compounds, which further create Fe^2+^ cations and OH^−^ radicals, using the absorbed UV/visible light energy, which is followed up by the transformation or mineralization of the pollutants through various reactions (De la Cruz et al. 2012). This process is quicker than the conventional Fenton process, and the recycling of the Fe^2+^ cations takes place at a higher rate. Alkyl radicals may also be created in a similar manner. Fe^3+^ cations precipitate through the formulation of amorphous ferric oxyhydroxides at higher pH levels, which makes it hard to recycle the Fe^2+^ cations (Ahmed et al. 2017). The whole process should thus take place at an optimal low pH level.

Molina et al. (2010) reported that iron loading has a more significant effect than the catalyst concentration, highlighting the significance of iron loading regarding the overall process efficiency. Iron concentration in the catalyst was in turn found to be more significant than the catalyst’s surface area (Domínguez et al. 2014).

Bautista et al. (2010) demonstrated that Fe/γ-Al_2_O_3_ is a very stable catalyst for the treatment of cosmetic wastewater over a period of 100 h. About 80% of chemical oxygen demand was removed at a temperature of 85 °C. The H_2_O_2_ was consumed in full, while the iron leaching over that time period remained below 3% of the starting iron weight.

**Fig. 7:**
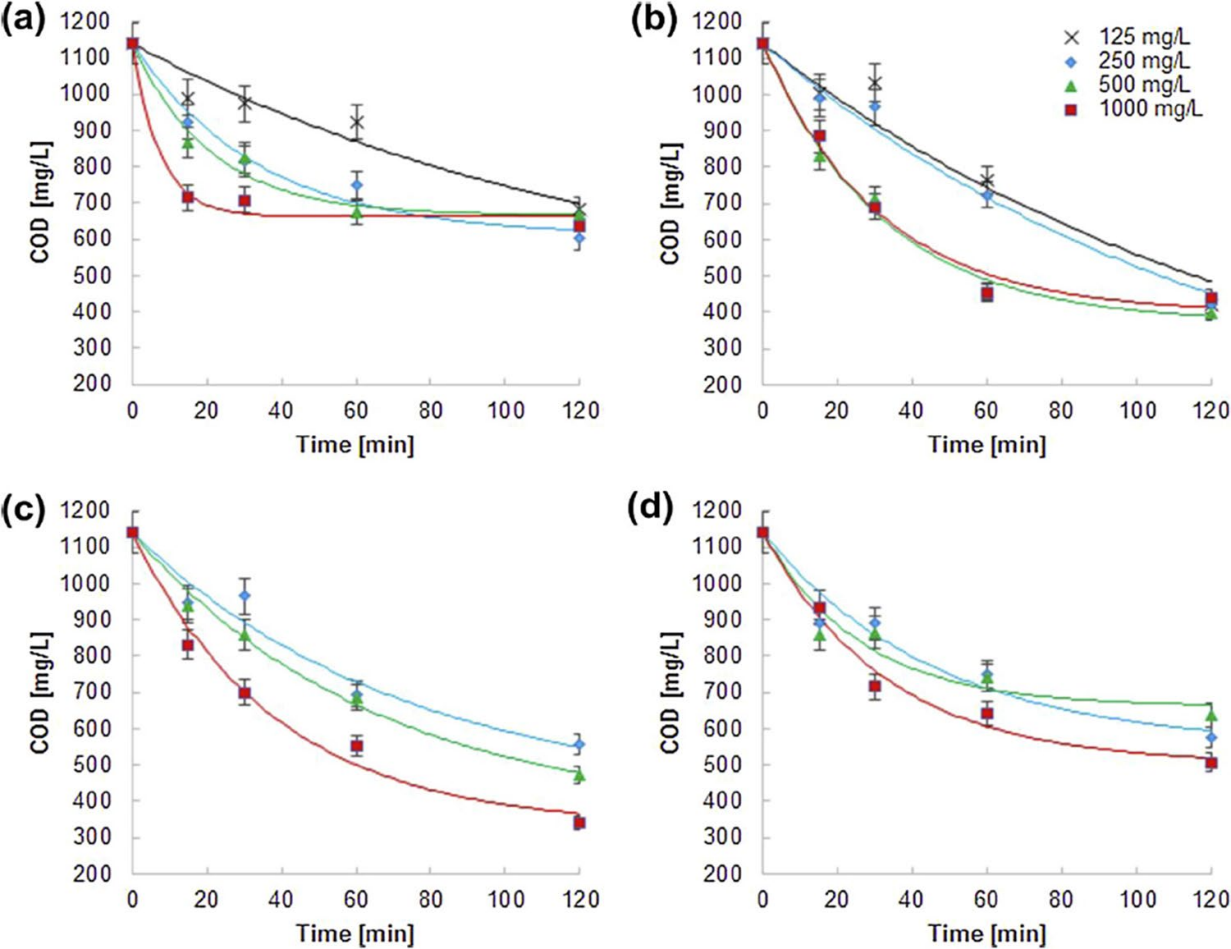
Chemical oxygen demand of CW after treatment by light/Fe^0^/H_2_O_2_ approach with various H_2_O_2_/chemical oxygen demand ratios: 0.5:1 (**a**),1:1 (**b**), 2:1 (**c**), and 4:1 (**d**) and various Fe.^0^ doses (125–1000 mg/L) (Muszyński et al. 2019)

The outcomes of the CW treatment through the light/Fe^0^/H_2_O_2_ process are presented in Fig. 6. Hydrogen peroxide was used in four different H_2_O_2_/COD mass ratios 0.5:1, 1:1, 2:1, and 4:1. Fe^0^ doses were reduced to 125, 250, 500, and 1000 mg/L, compared to the Fe^0^/H_2_O_2_ process (Fig. 7b) (Muszyński et al. 2019).

#### Cosmetics removal by chemical methods

A number of studies were performed to investigate chemical mechanisms of cosmetics removal (Table 7). The ozonation and photocatalysis mechanisms appear to be the most commonly used for the extraction of the evaluated compounds.

**Table 7:** Cosmetics removal by chemical methods

Compound	Material	Initial concentration	Removal (%, time)	Major mechanism	Ref
Methylparaben (MP), ethylparaben (EP), propylparaben (PP), butylparaben (BP)	Ozone	-	94.85–99.22% of all four simultaneously after 15 min	Ozonation and UV irradiation (O3/UV/TiO_2_/H_2_O)	Cuerda-Correa et al. (2016)
Methylparaben (MP), ethylparaben (EP), propylparaben (PP), butylparaben (BP), chemical oxygen demand COD	Ozone	70 mg H_2_O_2_/L, 8 mg O_3_/L	All parabens 100% after 120 min, COD 70%	O_3_/H_2_O_2_	Gmurek et al. (2019)
Methylparaben (MP), ethylparaben (EP), propylparaben (PP), butylparaben (BP), chemical oxygen demand COD	TiO_2_-Pt, TiO_2_-Pd, TiO_2_-Ag Ozone	45 mg O_3_	All parabens 100% after 120 min, COD 41–49%	O_3_/UVA/TiO_2_-Pt O_3_/UVA/TiO_2_-Pd O_3_/UVA/TiO_2_-Ag	Gmurek et al. (2019)
Chemical oxygen demand COD	Light/Fe^0^/H_2_O_2_	1000 mg/L Fe^0^ 2280 mg/L H_2_O_2_	70% after 120 min just by the combined process, then 97.7% after SBR	Combined light/Fe^0^/H_2_O_2_ and sequencing batch reactor (SBR)	Muszyński et al. (2019)
Total organic carbon (TOC)	H_2_O_2_/Fe_3_O_4_/Fe_2_O_3_/Fe^0^	500 mg/L Fe_3_O_4_ 500 mg/L Fe_2_O_3_ 1000 mg/L Fe^0^	56.2% after 120 min	UV/H_2_O_2_/Fe_3_O_4_/Fe_2_O_3_/Fe^0^	Bogacki et al. (2020)
Total organic carbon (TOC), chemical oxygen demand (COD)	Metallurgical waste, H_2_O_2_	MW 8.0 g/L, H_2_O_2_ 0.05 g/L	TOC 75% after 6 min COD 99% after 6 min	heterogeneous photo Fenton-Like degradation treatment	de Andrade et al. (2020)
Brilliant Green Methylene Blue	rGO/Ag_2_O Nanocomposite	30 mg in 15 mL MB or BG solution (10 ppm)	BG 75%, after 70 min MB 90%, after 150 min	photocatalytic reduction	Iqbal et al. (2021)

Cuerda-Correa et al. (2016) studied the elimination of members the parabens family (methylparaben (MP), ethylparaben (EP), propylparaben (PP), butylparaben (BP)) in ultrapure water through ozonation. Under optimal circumstances, a removal efficiency ranging between 95 and 99% was achieved. Direct ozonation was found to be the main degradation mechanism. Findings also suggest that single ozonation is a more efficient than the O_3_/H_2_O_2_ and O_3_/Fenton processes. Furthermore, using UV irradiation leads under all circumstances to a faster and more effective elimination of the parabens, due to the additional contribution of ozone photolysis. The optimal process for the degradation of these foulants was O_3_/UV/TiO_2_/H_2_O_2_.

Gmurek et al. (2019) explored an extensive comparison of various radical-driven technologies for paraben compound degradation. The assessment included (i) a comparison of ozone and peroxide processes; (ii) a comparison of catalytic and photocatalytic processes; (iii) the characterization of catalysts using various methods; (iv) an evaluation of the mineralization, biodegradability, and toxicity; and, lastly, (v) a cost. Photocatalysis treatments reduced both the chemical oxygen demand and the toxicity levels towards *Vibrio fischeri*, *Corbicula fluminea*, and *Lepidium sativum*, even though the full removal of chemical oxygen demand could not be ensured. The findings indicated that the treatment effectiveness and the related costs primarily depend on the implemented process. Titanium dioxide appears to be one of the most promising catalysts.

In a study by Muszynski et al., cosmetic wastewater was treated using a mix of light/Fe^0^/H_2_O_2_ process and biological treatment. The light/Fe^0^/H_2_O_2_ process achieved 70% removal of chemical oxygen demand after 2 h. The chemically treated wastewater went through biological treatment, leading to an overall chemical oxygen demand removal of up to almost 98%. These findings indicate the viability of combined AOP processes with bioremediation (Muszyński et al. 2019).

A study conducted by Jan Bogacki et al. used cosmetic wastewater that was treated with H_2_O_2_/Fe_3_O_4_/Fe_2_O_3_/Fe^0^ and UV/H_2_O_2_/Fe_3_O_4_/Fe_2_O_3_/Fe^0^. The findings indicated that a 56% removal of the total organic carbon was achieved after 2 h of treatment. The chromatographic analysis detected and identified the foulants in the wastewater, which were eliminated during the treatment processes. Any processes taking places at a pH level higher than 3 were not effective. The UV treatment was more efficient. The hypothesis regarding the accuracy and reproducibility of the findings was verified (Bogacki et al. 2020).

Pryscilla Martins de Andrade et al. evaluated the heterogeneous photo Fenton-like treatment, using the metal residue as a catalyst, as well as identifying potential organic compounds adsorbed by the chemical sludge. The process used metallurgical waste as a source of iron, for 6 min of treatment. Under these circumstances, the removal rate did not exceed 75% for the total organic carbon and 99% for the chemical oxygen demand removal. The analysis of the residue indicated that about 11% of the mass of the organic materials was still adsorbed onto the residue. The FTIR analysis of the solid sample indicated that the adsorbed organic compound is potentially paraffin, which matches the type of effluent released by the cosmetics industry (de Andrade et al. 2020).

Mehreen Iqbal et al. described the single-step preparation of an rGO/Ag_2_O nanocompound for wastewater treatment, which they later characterized using various methods. More specifically, XRD data verified the synthesis of the nanocomposite. The SEM analysis revealed that the carbon sheet was randomly connected with Ag_2_O, while the average particle size was estimated to be approximately 25 nm. The nanocomposite exhibited efficient catalytic reduction of 4-nitrophenol to 4-aminophenol and an outstanding photocatalytic activity for the degradation of methyl blue and brilliant green (Iqbal et al. 2021).

## Discussion

The traditional wastewater treatment mechanisms include physical, chemical, biological, or combinations thereof, such as coagulation, dissolved air flotation, adsorption, activated sludge, biodegradation, constructed wetlands, and advanced oxidation processes.

For sorption technique, many sorbent materials such as metal oxides, metal chalcogenides, zeolites, metal organic frameworks (MOFs), clays, polymers, as well as carbon materials including fullerenes, nanodiamonds, activated carbon, carbon nanotubes (CNTs), graphene, and its derivatives have been extensively explored to mitigate water pollution issues. Activated carbon (AC) holds the longest track record among the carbon-based materials in purification. Due to the varied quality and inconsistency on the grade of AC that can be generated from a wide range of raw sources, their adsorption performance in water treatment can be greatly affected. Another disadvantage of AC can be related to its highly energy-intensive activation process with large amount of heat energy required and its high tendency to experience pore blockage by larger pollutants within its pore structure, which could limit the diffusion of subsequent smaller contaminants (Yap et al. 2021).

Meanwhile, graphene and its derivatives appear to be rising stars in water purification. The use of advanced graphene sorbents is expected to reduce the alarming water pollution and deliver clean water globally, particularly in dealing with the removal of multipollutants in water. As accentuated by several critical reviews, an enormous knowledge gap still exists to link the surface chemistry and physicochemical properties of advanced graphene sorbents with sorption performance for multiple pollutant control in water purification. The biomimetic polydopamine (PDA) graphene aerogel was not only a promising adsorbent, but also a catalyst to tackle a broad class of water contaminants including oils, organic solvents, and dyes (Yap et al. 2021).

Another important category of nanomaterials are silica-based nanomaterials, which are widely used for removing HM ions owing to their non-toxicity and excellent surface characteristics. Zero-valent metal nanoparticles have demonstrated their ability in remediating a variety of pollutants in wastewaters. During the last two decades, micro- and nano-scaled magnetic particles have attracted attention as adsorbents for eliminating the biological molecules, organic pollutants, and heavy metal ions from water and wastewater. The major advantage with magnetic nanomaterials lies in their easy recovery after exhaustion from the treated solution by for an external magnetic field, as presented in one of the studies performed using magnetic mesoporous silica nanospheres for the removal of Pb^2+^, Hg^2+^, and Pd^2+^ (Kumar et al. 2021).

Persulfate-based AOPs are also considered to have potential for environmental remediation, with various heterogeneous catalysts offering the backbone to many wastewater purification methods. Contrary to other typical nanocatalyst heterogeneous systems, the immobilized-catalyst system is able to circumvent the separation problem to decrease scour and prevent aggregation by anchoring nanoparticles onto porous or large-particle carrier (Guo et al. 2022).

Hindrances to the biological treatment of cosmetic wastewaters stem from the appearance of detergents, surfactants, hormones, cosmetics, and pharmaceutical compounds. There are various research works denouncing the possibility that surfactants may noticeably hinder the biological treatment processes. Biological treatment technologies are typically more environmentally friendly and cheaper compared than the physicochemical treatments. Constructed wetland treatments exhibited enhanced PPCP extraction. With higher than 99% effectiveness, they have become the most frequently used alternative among all the available biological treatment options (Cheng et al. 2021). Constructed wetlands are also considered to be inexpensive due to a relatively low construction, operation, and maintenance costs (Wu et al. 2015). Constructed wetlands include free water surface, horizontal and vertical flows, and hybrid CW systems, and can be combined to take advantage of the benefits of diverse systems (Vymazal 2011). The removal of PPCPs in constructed wetlands has been found to be significantly affected by the physicochemical properties of the PPCPs (Vymazal 2011), as well as the configuration and operation of the wetland and the environmental conditions (Garcia-Rodríguez et al. 2014).

Chemical treatment methods include oxidation, photocatalytic degradation, and photo-Fenton treatment. Their elimination efficiency depends on the compound. It was noted that many PPCPs were inefficiently eliminated in wastewater treatment plants when using traditional activated sludge processes, and significant quantities of PPCPs were still detected in effluent and/or biosolids (Melvin and Leusch 2016). The adsorption of PPCPs using activated carbon (AC), graphene, graphene oxide (GO), and carbon nanotubes (CNTs) has provided promising results. However, there are various limitations to be overcome before their large-scale application: (1) The adsorption ability of AC requires improvement. (2) The cost of production is quite high. The cost of graphene on its own restricts its application; thus, further research is required to produce high-surface-area graphene at low costs. (3) It is crucial to improve the production technique of carbon nanotubes. Further effort should be put into developing simple and effective production methods for carbon nanotubes. Additional attention should be paid to the recycling and regeneration of AC, graphene, GO, and CNTs. Lastly, the combination of adsorbents and PPCPs could have toxic effects on aquatic environments; thus, more studies should be conducted regarding the interaction of adsorbents and PPCPs and their toxicity risks (Wang and Wang 2016).

Advanced oxidation processes using ozone, hydrogen peroxide, and Fenton (Fe^2+^/H_2_O_2_) have been found to be very effective for the elimination of PPCPs (Ghatak 2014), through the generation of hydroxyl radicals that can break down PPCPs oxidatively. AOP methods have however the drawback of high energy demands for various critical devices such as ozonizers, UV lamps, and ultrasonicators, which lead to increased operational costs (Comninellis et al. 2008).

Nevertheless, biodegradation is not always efficient for the elimination of contaminants in the environment. These limitations can be addressed through biological acclimation and bioaugmentation. Plósz et al. (2012) established an activated sludge modelling framework for xenobiotic trace chemicals, in order to assess the parameters that impact the extraction of diclofenac and carbamazepine in activated sludge.

### Issues related to waste management

The increased quantity of wastewater sludge is a worldwide concern due to the continuous population growth and requirement for appropriate sanitation in wastewater treatment plants (WWTPs). Sludge treatment and disposal processes are crucial for the protection of the environment, because the remaining various organic pollutants, metals, and pathogenic microorganisms might create health issues and thus need to be eliminated. A wide range of physical, chemical, and biological approaches has been established to limit or manage sludge production.

The most common options for the disposal of sludge are incineration, landfills, ocean-dumping, agriculture (directly or after composting), and for the production of cement, bricks and asphalt. The optimal strategy should consider the following: (i) the costs of gas scrubbing for air pollution control, (ii) the potential discharge of heavy metals into the environment, and (iii) the applicability of incineration in the case of large WWTPs or when the quality of sludge is not appropriate for land application. Sludge management requires large amounts of energy (and has environmental effects), with the cost of sludge treatment constituting approximately half of the total running expenses of WWTPs. Sludge disposal processes were deemed as responsible for about 40% of the total greenhouse gas emissions from WWTPs, which could be reduced if the concept of circular economy was introduced (Gherghel et al. 2019).

### Recovery of water from wastewater and its re-use

In the existing legislation regulating landfilling and land application in terms of their use as sludge disposal options, various researchers have explored the reuse and recycling of sludge as a potential environmentally sustainable alternative (Smol et al. 2015). To this end, the European Commission has stated that “if waste is to become a resource to be fed back into the economy as a raw material, then, much higher priority needs to be given to reuse and recycling.” Sludge reuse as resources for various industries poses a viable possibility of waste management, taking into account the circular economy concept (Eliche-Quesada et al. 2011; Gherghel et al. 2019). The reuse of sludge and/or ash sludge to produce construction material fits the circular economy concept and has the potential to address the significant sludge disposal problems. The recovery of enzymes and proteins from sludge through ultrasonification is also a promising option, but it has not yet tested at a larger scale, because of little research on the relative newness of the concept, limited research, and expensive process (Gherghel et al. 2019).

### Economic analysis of the waste treatment technologies

The concept of sustainable wastewater management has been heavily articulated and should be considered through a multidisciplinary perspective (Ćetković et al. 2022; Molinos-Senante et al. 2010), highlighting the need for an economic analysis, as suggested by various researchers.

Garrido et al. showed the significance of performing a quantitative comparison of the effectiveness of WWTPs that use various technologies, in an effort to assist managers in making informed decisions when picking the most suitable technology (Sala-Garrido et al. 2011). Leoneti et al. (2010) proposed a compromise to the conflict between efficiency and cost in terms of selecting the WTS, whereas Karimi et al*.* suggested a fuzzy analytical hierarchy process to enable the decision-making process (Karimi et al. 2011). Kalbar et al*.* deemed this process as the most important task faced by wastewater management experts (Kalbar et al. 2012). Molinos-Senante et al*.* have performed a holistic evaluation according to the sustainability aspects (Molinos-Senante et al. 2014). As reported by Zeng et al., the wastewater treatment alternatives are commonly considered according to the financial data that can be found in the feasibility report of the wastewater treatment project (WTP), while the options that require minimum capital and operation costs are selected without requiring a deep exploration of the technologies behavior under economics variation (Zeng et al. 2007). Sancho et al. emphasize the significance of obtaining detailed knowledge regarding each cost associated with the process, as well as a further analysis on comparative data for the various technological options, in order to guarantee serviceable information for future projects (Hernandez-Sancho et al. 2011).

According to Abidami et al., the coagulation methods are mostly utilized for the removal of colloidal material with the possibility to impart color and turbidity. The advantages of this approach over other physicochemical alternatives are its low cost and limited energy consumption (Bello et al. 2018). As described by Rey et al., one of the benefits of the catalytic wet peroxide oxidation (CWPO) process is the ability to be performed in ambient conditions and at a lower cost (Rey et al. 2009). For Fenton’s oxidation reaction, the little to none energy requirement to activate the Fenton’s reagent (H_2_O_2_ and iron salts (Fe^2+^) renders this method as preferable over many physicochemical solutions. Nevertheless, the higher the ferrous ion concentration, the higher the concentration of residual iron and sludge that goes above the allowable limit, which in turn incurs high removal costs (Bello et al. 2018). Gherghel et al. studied a wide range of approaches for the extraction of enzymes from activated sludge, including stirring with additives such as detergents and cation exchange resins, ultrasonication, and combinations of multiple processes. The recovery of enzymes and proteins from sludge through ultrasonification is also a promising option, but it has not yet tested at a larger scale, because of little research on the relative newness of the concept, limited research, and expensive process (Gherghel et al. 2019).

Zhang et al. pointed out that anaerobic digestion (AD) is considered to be an inexpensive option, since it allows for the recovery of energy in the form of methane, with limited environmental effects. In many countries, AD has already been applied extensively (Zhang et al. 2017). According to Yap et al., even though the ion exchange process is effective in regard to water treatment that generates no sludge, it still remains a process that is applicable to only a small number of pollutants, with a high cost for the replacement of the ion exchange resin long-term-wise. Nevertheless, water decontamination through membrane filtration can result in high removal efficiency via a simple separation process, without generating secondary pollution, although its application is still restricted by high production costs, high levels of fouling, and requirement of high energy consumption. Advanced oxidation process, on the other hand, emphasizes the use of strong oxidants or ultra-violet (UV) irradiation on a catalyst that often involves high operating cost with inefficient utilization of generated reactive oxygen species (Yap et al. 2021).

Lefebvre et al. argued that the use of microbial fuel cells (MFC) for electricity production is considered a sustainable solution for different problems such as excess sludge and water-energy crisis. Although the use of MFC technologies in WWTPs can improve the treatment performances, their application is limited due to the electrode materials that are expensive (Lefebvre et al. 2011). Gherghel et al. evaluated treatment technologies and reported that for the lowest TRLs; many challenges still exist and more studies are necessary, in terms of technology, costs, and environmental feasibility. The most promising technologies in the context of a circular economy are those for the recovery of phosphorus by struvite precipitation and energy by means of anaerobic digestion, thermal hydrolysis, and co-digestion with organic wastes. In fact, they are associated with the highest values of TRL. This also means that these technologies are immediately ready to penetrate the market and, as such, would radically change the current vision of a WWTP (Gherghel et al. 2019).

## Conclusions

It is difficult to adopt a universal treatment method that would be suitable for the removal of all contaminants from wastewaters. Selecting a suitable treatment method should thus rely on the wastewater characteristics.

Adsorption has been extensively studied as a cost-effective and environment-friendly method of cleaning wastewater. Physical approaches have been taken into account as pre-treatment options in along with other potential methods. The main drawback of this process is the membrane fouling. Chemical oxidation, particularly advanced oxidation processes (AOP), is currently at the research spotlight for water treatment. This type of process requires an active oxidation species (for ex •OH radicals), which would oxidize and mineralize the contaminated particles. Physical adsorption is a requirement for AOP, enabling the oxidation of contaminants on the surface of catalysts. As a result, the blend of physical and chemical processes appears to be an attractive wastewater treatment solution, especially versus organic contaminants.

Biological treatment of wastewater utilizes microorganisms that are able to degrade organic water pollutants, with oxygen/air being available or not (aerobic/anaerobic). It is often used for the production of fertilizers or nutrients for other organisms. It is cost-effective, environmentally-friendly, and does not need to be maintained often, thus becoming an accessible wastewater treatment option. It is notable, however, that the degradation process is time-consuming and requires a culture growth.

It is crucial to establish a physicochemical wastewater treatment system that mainly uses coagulation to augment the particles size of the product increases, through agglomeration of the particles into a larger size. After the coagulation, though, specific operating conditions must be met, to improve the efficiency of this process. Among the most significant conditions is the PH level, which relies on the form of the coagulant in the sewage tank. Additionally, the improvement of the coagulation calls for the use of a coagulant aid, specifically the biodegradable polyelectrolyte at the optimum dosage. To be able to assess the effectiveness of the coagulant used, it is important to determine the remaining concentration of the metallic ions in the treated wastewater after the process is completed. The use of a catalytic wet air oxidation is an alternative treatment method, through the oxidation of the contaminant with hydrogen peroxide in the presence of catalysts carrying metals. It should be stated that such a process is not optimal due to the limited PH range and the difficulty to recover the used catalyst, otherwise referred to as secondary pollution.

Each option has its unique advantages and limitations, not only cost-wise, but also in terms of viability, effectiveness, environmental impact, sludge production, and more. Currently, only a few of the cited processes have been effectively employed in a large scale due to economic reason. This review described the available options for the cosmetic wastewater treatment technologies, noting, however, that only few of them are actively used on a large scale, due financial and technological limitations.

## Supplementary Information

Below is the link to the electronic supplementary material.

Supplementary file1 (rar 413 KB)

## Data Availability

Not applicable.
